# IAPP modulates cellular autophagy, apoptosis, and extracellular matrix metabolism in human intervertebral disc cells

**DOI:** 10.1038/cddiscovery.2016.107

**Published:** 2017-01-30

**Authors:** Xinghuo Wu, Yu Song, Wei Liu, Kun Wang, Yong Gao, Shuai Li, Zhenfeng Duan, Zengwu Shao, Shuhua Yang, Cao Yang

**Affiliations:** 1 Department of Orthopaedic Surgery, Union Hospital, Tongji Medical College, Huazhong University of Science and Technology, Wuhan 430022, China; 2 Department of Orthopaedic Surgery, Massachusetts General Hospital and Harvard Medical School, Boston, MA, USA

## Abstract

The pathogenic process of intervertebral disc degeneration (IDD) is characterized by imbalance in the extracellular matrix (ECM) metabolism. Nucleus pulposus (NP) cells have important roles in maintaining the proper structure and tissue homeostasis of disc ECM. These cells need adequate supply of glucose and oxygen. Islet amyloid polypeptide (IAPP) exerts its biological effects by regulating glucose metabolism. The purpose of this study was to investigate the expression of IAPP in degenerated IVD tissue, and IAPP modulation of ECM metabolism in human NP cells, especially the crosstalk mechanism between apoptosis and autophagy in these cells. We found that the expression of IAPP and Calcr-RAMP decreased considerably during IDD progression, along with the decrease in the expression of AG, BG, and Col2A1. Induction of IAPP in NP cells by transfection with pLV-IAPP enhanced the synthesis of aggrecan and Col2A1 and attenuated the expression of pro-inflammatory factors, tumor necrosis factor (TNF)-*α*, and interleukin (IL)-1. Upregulation of IAPP also affected the expression of the catabolic markers—matrix metalloproteinases (MMPs) 3, 9 and 13 and ADAMTS 4 and 5. Downregulation of IAPP by siRNA inhibited the expression of anabolic genes but increased the expression of catabolic genes and inflammatory factors. The expressions of autophagic and apoptotic markers in NP cells transfected with pLV-IAPP were upregulated, including BECLIN1, ATG5, ATG7, LC3 II/I and Bcl-2, while significantly increase in the expression of Bax and Caspase-3 in NP cells transfected with pLV-siIAPP. Mechanistically, PI3K/AKT-mTOR and p38/JNK MAPK signal pathways were involved. We propose that IAPP might play a pivotal role in the development of IDD, by regulating ECM metabolism and controlling the crosstalk between apoptosis and autophagy in NP, thus potentially offering a novel therapeutic approach to the treatment of IDD.

## Introduction

Intervertebral disc degeneration (IDD) is a common cause of low back pain. The pathogenic processes of IDD include biochemical, cellular, and structural disorders of inter vertebral discs (IVD) that exacerbate metabolic imbalance of the extracellular matrix (ECM).^1–3^ Increased cell death in the disc has been demonstrated to contribute to the process of IDD.^4,5^ It is known that impaired IVD cells can cause an imbalance in these processes, leading to disc degenerative changes.^6,7^ Disc cells require essential nutrients such as glucose and oxygen for optimal cellular activity and function. Any change in the nutrient balance in the disc affects cellular activity and cell survival significantly.^8,9^

Islet amyloid polypeptide (IAPP) is a 37-residue peptide hormone, which is involved in the regulation of glucose homeostasis.^10^ In addition to its function in islet cells, IAPP has also been shown to function as insulin in peripheral tissues. IAPP exerts its biological function through the signaling pathway that includes G-protein-coupled calcitonin receptor (Calcr) and associated receptor activity modifying proteins (RAMPs; RAMP1–3),^11^ which have been detected in pancreas and many extra-pancreatic tissues, such as central nerve system (CNS), liver, kidney, muscle, and adipose tissue.^12–16^ Specifically, IAPP acts as a neuroendocrine regulator to control glucose metabolism, or as a growth factor in extra-pancreatic tissues. In addition, previous studies revealed that human IAPP (hIAPP) has the function of promoting the secretion of IL-1*β*, IL-1*α*, and TNF-*α* by activating macrophages *in vitro,*^17,18^ all of which have been shown to be closely associated with IDD through degradation of ECM.^19–21^ If IAPP has a predominant role in IVD degeneration, it may be a good target for designing successful therapies.

Autophagy, apoptosis, and senescence of the cells are distinct cellular processes that account for disc degeneration.^22–24^ It is becoming increasingly apparent that apoptosis and autophagy are closely linked but mutually exclusive processes, with complementary pathways involved in in the pathogenesis of IDD.^25,26^ Apoptosis is closely associated with and shares molecular events with autophagy in the pathogenesis of human diseases.^27^ Autophagy is an essential intracellular protein degradation system and contributes to intracellular quality control by clearing damaged organelles and damaged proteins.^28,29^ Therefore, it is essential and necessary to investigate the crosstalk mechanism of autophagy and apoptosis in the IVD cells during the pathogenesis of IDD. The aim of this study was to determine whether IAPP is involved in the development of IDD, especially in the anabolic and catabolic pathways of ECM, and the possible mechanisms.

## Results

### IAPP gene expression in normal and degenerated NP tissue

**Figure 1:**
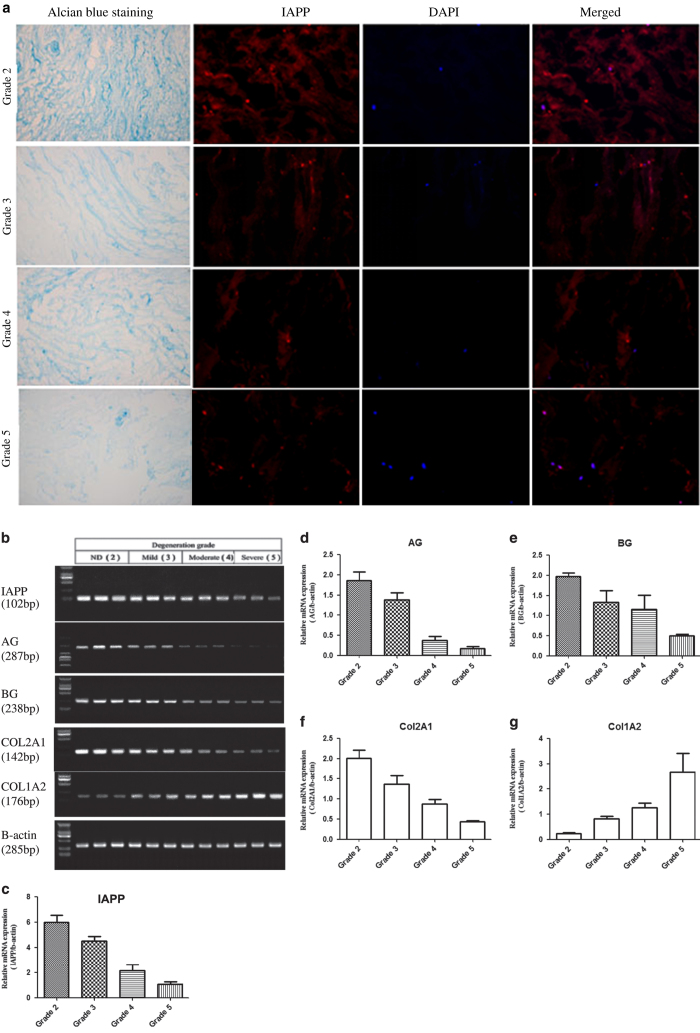
Detection of IAPP expression in human IVD tissue. (**a**) Representative images of IAPP protein in human IVD tissue detected by immunofluorescence staining. (**b**) IAPP, AG, BG, and Col mRNA levels in human IVD tissue samples were determined by RT-qPCR. (**c**) Statistical analysis showing the relative expression of IAPP mRNA in IVD tissue with varying degrees of degeneration. (**d**–**g**) Statistical analysis showing the relative expression of AG (**d**), BG (**e**), Col2A1 (**f**), and Col1A2 (**g**) mRNAs. Data were shown as means±S.D. *P<0.05, **P<0.01, compared with group 2 (non-degenerated IVD). Grades 3, 4, and 5 signify mild, moderate, and severe degeneration, respectively. (Alcian blue staining ×100; IHC ×200).

The degree of degeneration of the IVD tissue was classified using MRI images and confirmed by Alcian blue staining (Figure 1a). Lower expression of IAPP corresponded with higher degenerative degree (Figure 1a). IAPP mRNA expression levels in human IVD tissue, normalized with respect to *β*-actin, are presented as 2^-ΔΔ^Ct (Figure 1b). As can be seen in Figure 1c, IAPP expression in normal IVD tissue (Grade 2) was significantly higher compared with that in degenerated disc tissue. Proteoglycans and collagens are the main components of ECM. During the process of IDD, the gene expression of AG, BG, and collagen (Col)2A1 were decreased (Figures 1d–f), whereas the expression of Col1A2 was increased (Figure 1g), which confirmed disc degeneration. Thus, the results demonstrated that the gene expression of IAPP decreased during the process of disc degeneration.

### IAPP, Calcr, and RAMP3 expression in NP cells

**Figure 2:**
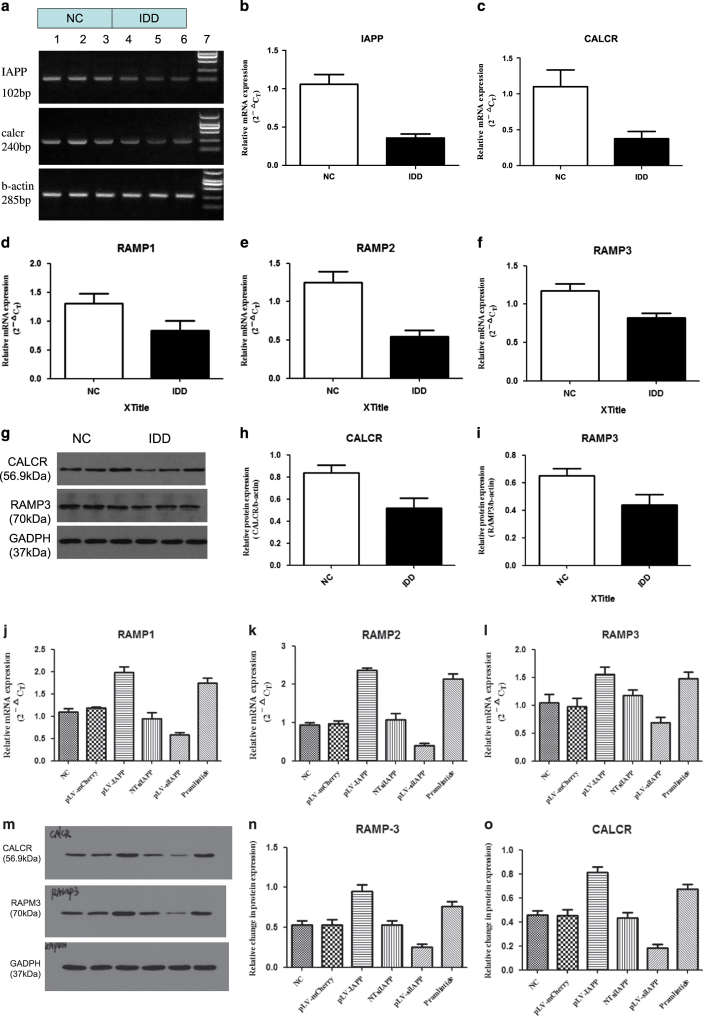
Detection of the expression of IAPP and its receptors in NP cells. (**a**) IAPP and Calcr mRNA levels in normal and degenerated IVD NP cells were determined by RT-qPCR. (**b**) Statistical analysis showing mRNA expression of IAPP in different groups. (**c**) Statistical analysis showing mRNA expression of Calcr. (**d**–**f**) Statistical analysis showing mRNA expression of RAMP1, RAMP2, and RAMP3. (**g**) Protein expression of Calcr and RAMP3 in NP cells was determined by western blotting. (**h**) Statistical analysis showing the relative expression of Calcr protein. (**i**) Statistical analysis showing the relative expression of RAMP3 protein. (**j**–**l**) Statistical analysis showing mRNA expression of IAPP in NP cells infected with pLVX-mCMV-Puro, pLVX-mCMV-IAPP, PLVX-ShRNA2-Puro, pLVX-ShRNA2- Puro-IAPP, or treated with pramlintide. (**m**) Protein expression of Calcr and RAMP3 in NP cells of different groups were determined by western blotting. (**n**) Statistical analysis showing the relative expression of RAMP3 protein. (**o**) Statistical analysis showing the relative expression of Calcr protein. Data were shown as means±S.D. *P<0.05, **P<0.01, compared with the normal group.

RT-PCR analysis was used to detect the mRNA expression of IAPP, Calcr, and RAMP3 (Figure 2a). The results showed that mRNA expression of IAPP and Calcr was significantly lower in degenerated group compared to that in normal group (Figures 2b and c). The gene expressions of RAMP1,2,3 were all lower in the degenerated group, compared to those in the normal group (Figures 2d–f). Cell lysates from both groups were examined by western blot, which also confirmed a significant decrease of Calcr and RAMP3 protein expressions in degenerated NP cells (Figures 2g–i). The results indicated that the protein expression levels of Calcr and RAMP3 were also lower and consistent with the decreased expression of IAPP (Figures 2j–o). Knockout of IAPP gene expression resulted in lower expression of Calcr and RAMP3 receptors. Pramlintide also promoted the expression of Calcr and RAMP3 receptors. Thus, Calcr and RAMP3 receptors are required for secreted IAPP to suppress IVD degeneration.

### IAPP regulates anabolic and inflammatory cytokines in NP cell metabolism

**Figure 3:**
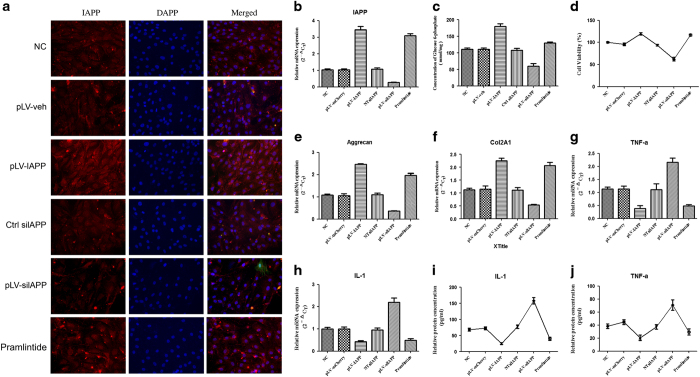
The effects of IAPP on ECM anabolic gene expression and pro-inflammatory factor secretion in NP cells. Immunocytochemistry assay revealed IAPP protein expression in NP cells infected with pLVX-mCMV-Puro, pLVX-mCMV-IAPP, PLVX-ShRNA2-Puro, pLVX-ShRNA2-Puro-IAPP, or treated with pramlintide. (**a**) Immunocytochemistry assay revealed IAPP protein expression in NP cells after transfection. (**b**) RT-PCR showed the expression of IAPP in NP cells in different groups. (**c**) MTT assay showed higher cell viability resulting from the upregulation of IAPP. (**d**) Glucose uptake measurement. E-H. RT- PCR showed gene expression of aggrecan (**e**), Col2 (**f**), and downregulation of TNF-*α* (**g**) and IL-1 (**h**) in different groups. (**i** and **j**). ELISA analysis showed protein expression of IL-1 (**i**) and TNF-*α* (**j**) in human NP cells of different groups. *P<0.05, **P<0.01, compared with NC.

As shown in Figures 3a and b, the expression of IAPP was enhanced in pLV-IAPP group and decreased in pLV-siIAPP group, compared to that in control group. Upregulation of IAPP resulted in an increase in glucose uptake (Figure 3c) and cell proliferation in cultured NP cells (Figure 3d). Knockdown of IAPP resulted in lower expression of aggrecan and Col2A1 and higher expression of TNF-*α* and IL-1, whereas upregulation of IAPP resulted in higher expression of aggrecan and Col2A1 and lower expression of TNF-*α* and IL-1, compared to in thencontrol group (Figures 3e–j). We treated NP cells with pramlintide, a synthetic analog of amylin, which exerted similar effects as pLV-IAPP.

### IAPP regulates ECM catabolic gene expression in NP cells

**Figure 4:**
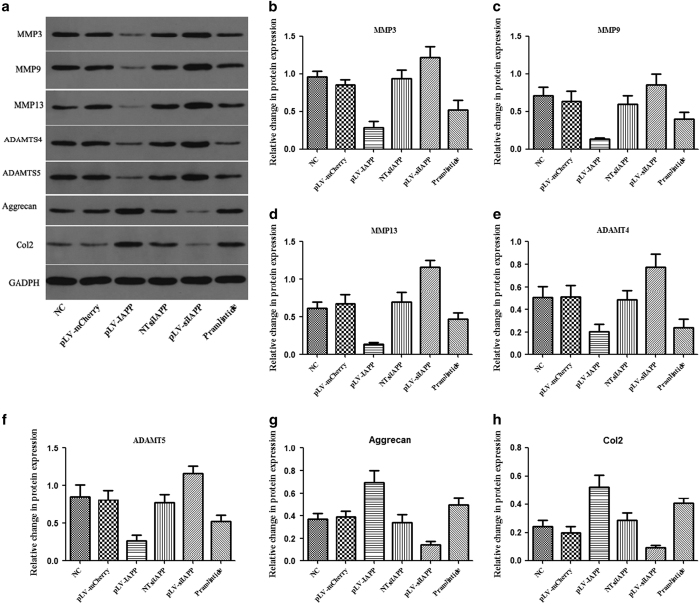
Effects of IAPP on the expression ECM catabolic genes in human NP cells. The cells were transfected with pLVX-mCMV-Puro, pLVX-mCMV-IAPP, PLVX-ShRNA2-Puro, pLVX-ShRNA2-Puro-IAPP, or treated with pramlintide. (**a**) Protein expressions of MMP-3, MMP-9, MMP-13, ADAMTS-4, and ADAMTS-5 were determined by western blotting. (**b**–**h**) Statistical analysis showing the relative expression of MMP-3 (**b**), MMP-9 (**c**), MMP-13 (**d**), ADAMTS-4 (**e**), ADAMTS-5 (**f**), Aggrecan (**g**), and Col2 (**h**) proteins. Values represent the means±S.D. *P<0.05, **P<0.01, compared with NC.

To better understand the effects of IAPP on ECM metabolism in NP cells, we knocked down the expression of IAPP using siRNA and examined the catabolic gene expression of MMP-3,9,13, and ADMTS-4 and 5 by western blotting. As expected, expression of the ECM catabolic enzymes MMP-3, 9, 13 and ADAMTS-4 and 5 were strongly increased (Figures 4a–f). Upregulation of IAPP resulted in a significant decrease in the basal protein levels of these catabolic enzymes, and increased levels of Aggrecan and Col2 proteins (Figures 4g and h). These results indicate that IAPP is involved in the *in vitro* disc degenerative effects mediated by MMPs. Pramlintide also inhibited the expression of MMPs.

### Apoptotic and autophagic gene expression in NP cells regulated by IAPP

**Figure 5:**
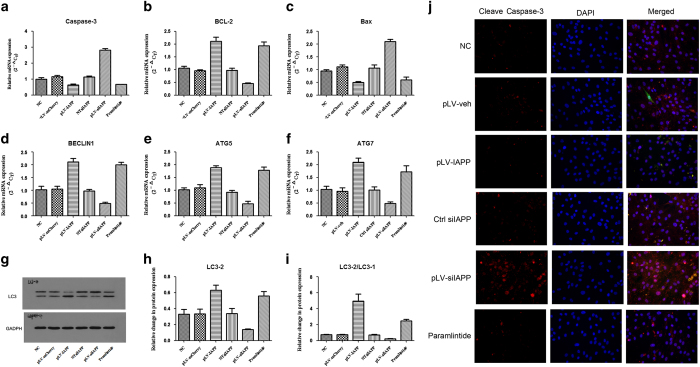
Effects of IAPP on apoptotic and autophagic gene expression in NP cells. NP cells were transfected with pLVX-mCMV-Puro, pLVX-mCMV-IAPP, PLVX-ShRNA2-Puro, pLVX-ShRNA2-Puro-IAPP, or treated with pramlintide. (**a**–**c**) RT- PCR analysis was used to detect the mRNA expression of apoptotic genes, including caspase-3 (**a**), Bcl-2 (**b**), and Bax (**c**). (**d**–**f**) RT- PCR analysis was used to detect the mRNA expression of autophagic genes, including BECLIN1 (**d**), ATG5 (**e**), and ATG7 (**f**). (**g**) Protein expression of LC-3 in different groups of NP cells was determined by western blotting. (**h** and **i**) Statistical analysis showing the relative expression of LC3-2 (**h**) protein and the ratio of LC3-2/LC3-1 (**i**). (**j**) Representative images of caspase-3 protein expression detected by immunofluorescence staining. Data were shown as means±S.D. *P<0.05, **P<0.01, compared with the NC. (Scale bar magnification: ×400).

Upregulation of IAPP resulted in increased expression of anti-apoptotic Bcl-2 mRNA and decreased expression of pro-apoptotic Bax and caspase-3 mRNAs. Suppression of IAPP expression by pLVX-ShRNA2-Puro-IAPP exerted opposite effects of increasing pro-apoptotic Bax and caspase-3 gene expression (Figures 5a–c). Upregulation of IAPP also increased the expression of autophagy genes, such as BECLIN1, ATG5, and ATG7. Suppression of IAPP expression could inhibit the expression of the autophagy genes (Figures 5d–f). Immunofluorescence assay for the detection of the accumulation of cleave caspase-3 protein in NP cells demonstrated that caspase3-positive NP cells were more apparent in pLV-siIAPP group (Figure 5j). The results of western blot analysis to detect the protein expression of LC-3 indicated that upregulation of IAPP could increase the expression of LC3-2 and the ratio of LC3-2/LC3-1(Figures 5g–i). LC3 is a known autophagy marker and the increased ratio of LC3-2/LC3-1 indicates that autophagy can be intensified by IAPP.

### Effects of IAPP on ROS content and apoptosis in NP cells

**Figure 6:**
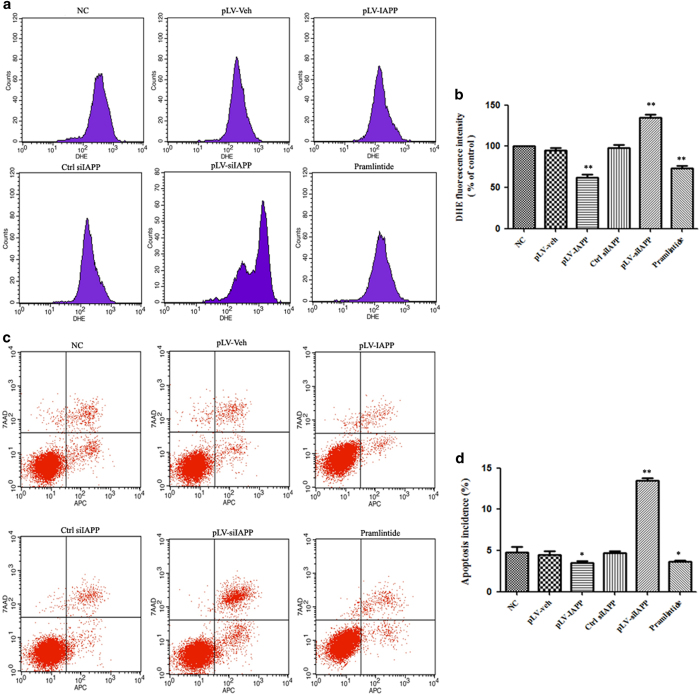
ROS content and apoptosis induced by IAPP were determined by Flow Cytometry. (**a**) Intracellular ROS generation was determined by measuring the fluorescence intensity using flow cytometry in NP cells infected with pLVX-mCMV-Puro, pLVX-mCMV-IAPP, PLVX-ShRNA2-Puro, pLVX-ShRNA2- Puro-IAPP, or treated with pramlintide. (**b**) Statistical analysis showing results of cell fluorescence for DHE. (**c**) Flow cytometric measurement for apoptosis incidence. (**d**). Statistical analysis showing TUNEL-positive rate. For quantitative evaluations, data were shown as means±S.D. *P<0.05, **P<0.01, compared with the NC.

To examine the effect of IAPP on intracellular accumulation of free radicals, ROS generation was analyzed by DCFH-DA using flow cytometry (Figure 6a). There was significant decrease in intracellular ROS by the upregulation of IAPP in NP cells transfected with pLV-IAPP, compared to in the NC group. Downregulation of IAPP by siIAPP could induce an obvious ROS burst in NP cells (Figure 6b). The level of apoptosis in NP cells was measured by flow cytometric analysis, which demonstrated that the apoptosis level was much higher in NP cells transfected with pLV-siIAPP, compared with that in NP cells transfected with pLV-IAPP (Figures 6c and d).

### Induction of apoptosis and autophagy in NP cells by IAPP

**Figure 7:**
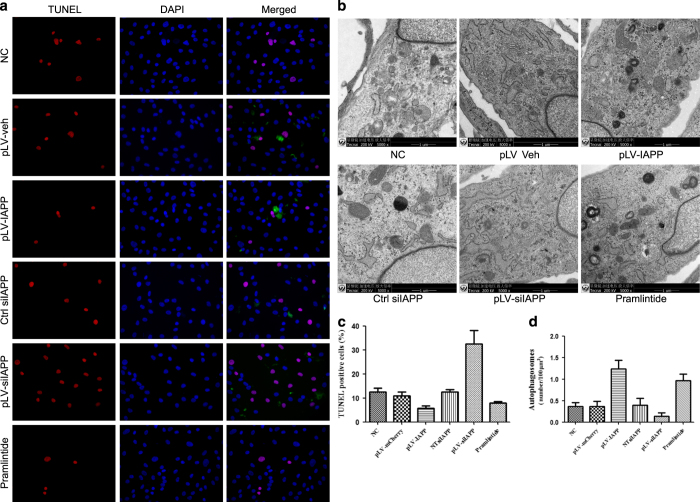
Apoptosis and autophage Assay in NP cells induced by IAPP. (**a**) TUNEL assay to identify apoptotic cells (red) in NP cells transfected with pLVX-mCMV-Puro, pLVX-mCMV-IAPP, PLVX-ShRNA2-Puro, pLVX-ShRNA2- Puro-IAPP, or treated with pramlintide. (Scale bar magnification: ×400) (**c**). Statistical analysis showing results of apoptosis incidence. (**b**) EM analysis showed autophagosome formation in NP cells of different groups. Scale bar: 1 *μ*m. (**d**) Statistical diagram showing autophagosome counts. Values represent the means±S.D. *P<0.05, **P<0.01, compared with NC.

The level of apoptosis in NP cells was confirmed by TUNEL Assay. As shown in Figure 7a, a few apoptotic cells were stained red in normal NP cells, but apoptosis was much higher in NP cells transfected with pLV-siIAPP. TUNEL-positive cell proportion was much lower in NP cells transfected with pLV-IAPP and much higher in NP cells transfected with pLV-siIAPP, compared with in the normal group (Figure 7c). Pramlintide administration could also decrease the apoptotic rate. To detect the regulatory effect of IAPP on cell autophagy, NP cells were infected with pLV-si IAPP or pLV-IApp. Consistent with the expression of autophagic gene, overexpression could induce autophagosome accumulation in NP cells, while downregulation of IAPP could induce a decrease in autophagosome accumulation (Figures 7b and d). Thus, IAPP could mediate autophagy and decrease apoptosis to the rescue NP cells.

### Activation of PI3K/Akt-mTOR and P38/JNK MAPK signaling by IAPP induction

**Figure 8:**
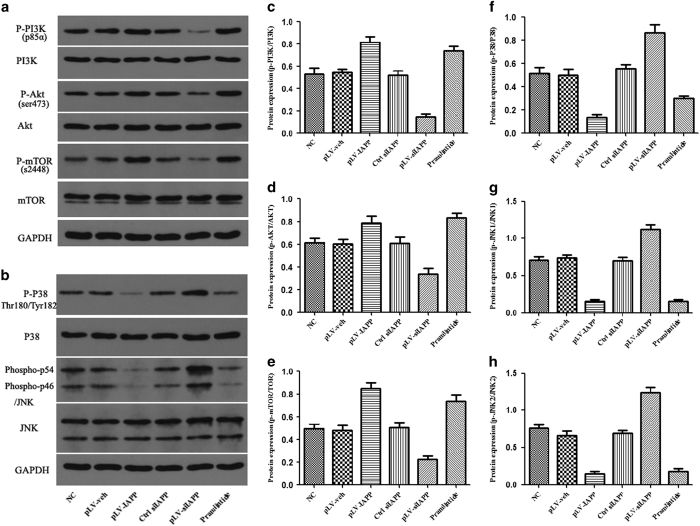
Effects of IAPP on the expression of PI3K/AKT-mTOR and p38/JNK MAPK signaling. (**a**) Western blot analysis revealed the phosphorylated and unphosphorylated protein levels of PI3K/AKT-mTOR signaling pathway. (**b**) Western blot analysis revealed the phosphorylated and unphosphorylated protein levels of P38 and JNK. (**c**–**e**) Statistical analysis of the ratio of P-PI3k/PI3K (**c**), p-AKT/AKT (**d**), and p-mTOR/mTOR (**e**) in different groups, including NC, pLV-veh, pLV-IAPP, Ctrl siIAPP, pLV-siIAPP, and Pramlintide. (**f**–**h**). Statistical analysis of the ratio of P-P38/P38 (**f**), p-JNK1/JNK1(**g**), p-JNK2/JNK2 (**h**) in different groups. Data are representative of three independent experiments, and *P*-values are shown: *P<0.05; **P<0.01.

The results showed that the activation of PI3K/Akt-mTOR was significantly enhanced in NP cells transfected with pLV-IAPP and IAPP siRNA administration could remarkably weaken the effects (Figure 8). MAPK signaling includes p38JNK, which is a key pathway regulating cell proliferation and apoptosis. To further identify the related pathway, we examined the p38/JNK MAPK signaling. The results of western blot analysis showed that IAPP treatment activates p38/JNK MAPK signaling by inhibiting the protein expression of phospho-p38 and phospho-JNK, whereas downregulation of IAPP by siRNA administration could inactivate p38/JNK MAPK signaling, resulting in increased expression of phospho-p38 and phospho-JNK. Therefore, the PI3K/Akt-mTOR signaling and p38/JNK MAPK signaling pathways are coordinately involved in the IAPP mediated regulation of NP cell metabolism.

## Discussion

IDD is a progressive phenomenon that begins in the NP with a decrease in proteoglycans.^3^ Progression of the degeneration in the late stages impacts collagen synthesis and degradation leading eventually to disc collapse,^30^ and increased mechanical load on the vertebral body.^31^ Therefore, the treatment of disc degeneration should focus on the homeostasis of the ECM in the disc. The NP cells have a pivotal role in ECM metabolism, providing activated substrates for proteoglycan anabolic and catabolic pathways.^32–34^ The results of our studies showed that the gene expression of AG, BG and Col2A1 were decreased significantly during the process of IDD, accompanied by a decrease in IAPP expression. IAPP exerts its effects in NP cells via calcitonin and RAMP receptors. Decreased expression of IAPP in degenerated NP cells results in the inhibition of Calcr and RAMP3 protein expression, which was confirmed by the transfection of NP with pLV-siIAPP.

Matrix metalloproteinases (MMPs) are a growing family of metalloendopeptidases that degrade some of the extracellular matrix components. Upregulated MMP and ADAMTS cause pathological proteolysis of disc ECM and the subsequent development of IDD.^35,36^ The inflammatory cytokine IL-1*β* is a widely used key factor in regulating the expression of MMPs of disc cells, therefore, it is used to mimic the catabolic state of NP cells. Upregulation of IAPP in NP cells transfected with pLV-IAPP resulted in lower expression of MMP-3,9,13 and ADAMTS-4,5. Knocking down IAPP expression in NP cells by transfection with pLV-siIAPP significantly increased the expression of these proteolytic enzymes Thus, IAPP has an important role in MMPs-induced IDD. Similar to chondrocytes, NP cells are sensitive to apoptosis induced by IL-1*β*. In contrast, IL-1*β* alone does not induce significant apoptotic changes in AF cells. The results of this study indicated that abnormal expression of IAPP in the NP cellstriggers secretion of IL-1 and TNF-*α*, which act as pro-inflammatory cytokines that regulate MMP expression and accelerate disc degeneration.

Apoptosis and autophagy are two patterns of programmed cell death, which could decrease the number of viable IVD cells and accelerate the degenerative process.^4,24^ Apoptosis can be induced in IVD cells in animal models of human disc degeneration, and was increased in diabetic rats.^37,38^ The NP cells exposed to excessive glucose showed decreased proliferation and increased apoptosis in rat model.^39^ In this study, IAPP K/D cells were growing in culture without a stimuli (e.g., serum starvation), however, the apoptotic rate was significantly higher in IAPP K/D NP cells, compared with control groups. We found that the downregulation of IAPP in NP cells transfected with pLV-siIAPP resulted in decreased cell proliferation, increased expression of pro-apoptotic Bax and Caspase-3, and intensive TUNEL staining.

Autophagy is a protective mechanism in IVD, degrading excess or damaged proteins and dysfunctional organelles associated with disc degeneration.^25^ Although the exact mechanism is not clear, some genes have been found to be closely linked to the formation and maturation of autophagosome, such as beclin-1 and microtubule-associated protein 1 light chain 3 (LC3).^40–42^ During autophagy, a cytosolic form of LC3 (LC3-I) is lipidated and converted to LC3-phosphatidylethanolamine conjugate (LC3-II), which is recruited to autophagosomal membranes.^43,44^ Thus, LC3-I conversion to LC3-II and accumulation of LC3-II are widely used as reliable autophagosomal markers for monitoring autophagy and autophagy related processes. The increased level of LC3-II/LC3-I, beclin-1, ATG5, and ATG7 indicated the increase of autophagy activity in NP cells transfected with pLV-IAPP. Our studies indicated that autophagy served as an important protective mechanism in human degenerative diseases through the cellular catabolic pathways that included lysosomal degradation and recycling of proteins and organelles, and is regulated by IAPP.

Phosphoinositide-3 kinase (PI3K)/AKT signaling regulates several important functions in cells, such as cell growth, proliferation, migration and apoptosis.^45–47^ Upregulation of IAPP promoted PI3K expression in our experiments, indicating that PI3K/AKT pathway mediated the role of IAPP in regulating apoptosis of NP cells. mTOR is a downstream effector of the PI3K/AKT pathway, and activated mTOR inhibits the process of autophagy.^48–50^ Mitogen-activated protein kinases (MAPK) are activated in response to a variety of extracellular signaling and participate in cell proliferation, differentiation, apoptosis, and cellular stress responses.^51^ The activation of p38 and JNK MAPK pathway is often correlated with inhibited protein expression of p38 and JNK to prevent apoptosis and induce autophagy.^52,53^ Our results showed that overexpression of IAPP significantly upregulated the phosphorylation of AKT and mTOR, downregulated the expression of phosphorylated-p38 and JNK and the gene expression of Bcl-2, BECLIN1, ATG5, and ATG7, thereby increasing autophagy and decreasing apoptosis in NP cells. When the expression of IAPP was inhibited by siRNA, PI3K/AKT-mTOR and p38/JNK MAPK signaling was suppressed; expression of caspase-3 and Bax increased; and expression of BECLIN1, ATG5, and ATG7 decreased. Thus, apoptotic effect was enhanced and the autophagic activity was weakened in NP cells transfected with siIAPP. These results confirmed that the PI3K/AKT/mTOR and p38/JNK MAPK signaling was involved in IAPP mediated effects.

The results presented in this study provide new insights and help us understand why disc degeneration is more prevalent in those with inadequate nutrition. IAPP regulates glucose uptake, ECM anabolic and catabolic gene expression, controls the crosstalk between apoptosis and autophagy through PI3K/AKT/mTOR and p38/JNK MAPK signaling in NP cells. During IDD, the expression of IAPP, Calcr, and RAMP3 was decreased, which aggravates IDD through the mechanism described above. Pramlintide, a synthetic analog of amylin, can regulate the secretion of IAPP and improve cell growth, proliferation, autophagy, and apoptosis of NP cells. Therefore, it could be a novel drug candidate for the treatment of IDD.

## Materials and Methods

### Tissue selection and grading of IVDs

This study has been approved by the Ethics Committee of Tongji Medical College, and written informed consents have been obtained. Human IVD tissue was obtained at surgery. Patients were selected on the basis of MRI determination of disc degeneration requiring disc excision and spinal fusion surgery for IDD (*n*=32) or deformity correction surgery for scoliosis (*n*=8). The IVD cryosections from each group were stained with Alcian blue.

### Isolation and culture of NP cells

Human tissue samples were obtained with informed consent of the patients and ethical committee approval. The IVD tissues were collected during surgery and transferred to the laboratory within 60 min. Briefly, NP tissues were minced, enzymatically digested in 0.2% type II collagenase and 0.25% trypsin for 3 h, and filtered to obtain single-cell suspensions. The cells were seeded and cultured in DMEM/F-12 containing 20% fetal bovine serum, 50 U/ml penicillin, and 50 *μ*g/ml streptomycin at 37 °C in a humidified (5% CO2, 95% air) incubator. The cells were passaged two to three times before being used for the subsequent experiments.

### Immunohistochemistry

Sections of 5 *μ*m thickness were cut from paraffin-embedded tissues. The sections were deparaffinized in xylene and rehydrated in a gradient of ethanol. After washing, endogenous peroxidase activity was blocked by immersing the slides in 0.3% H_2_O_2_ at room temperature. The sections were incubated with polyclonal anti-IAPP (1 : 100), or control rabbit IgG (2 *μ*g/ml) at 4 °C overnight. The sections were incubated for 1 h at room temperature with secondary antibodies, goat anti-rabbit fluorescein-conjugated antibody (1 : 200). Nuclei were visualized by staining with DAPI (Beyotime Biotechnology (Shanghai, China), 0.5 g/ml) and images were captured using fluorescence microscope (Olympus, Takachiho, Japan).

### Viral transfection of NP cells *in vitro*

The NP cells were seeded and cultured at a density of 2×10^5^ cells in six-well dishes and allowed to reach 70–80% confluence before infection. After discarding the medium from each well and making the total volume up to 1 ml, NP cells were infected with pLVX-mCMV-ZsGreen-PGK-Puro, pLVX-mCMV-ZsGreen-PGK- Puro-IAPP, PLVX-ShRNA2-Puro, pLVX-ShRNA2-Puro-IAPP, or treated with pramlintide (peprospec, HOR-300). Homo IAPP Gene ID: 3375, and shRNA plasmids for IAPP: sequence 5′-GCTGCAAGTATTTCTCATT-3′. The cells were infected at a multiplicity of infection of 10^4^ vg/ml. Six hours later, the culture medium was added up to 2 ml. Transgenic expression in NP cells was detected using real-time PCR (RT-PCR) and western blot analysis at 48 h after transfection. The efficiency of transfection was quantified by assessing ZsGreen-positive cells under fluorescence microscope (Abcam, Cambridge, UK).

### MTT assay

The MTT assay is a colorimetric assay for assessing cell proliferation. The supernatant was aspirated from each well after 72 h, 10 *μ*l MTT was added to each well and incubated for 4 h at 37 °C. The medium from each well was aspirated, 150 *μ*l DMSO was added to each well and the plates were agitated for 10 min in a shaker at 700 r.p.m. Finally, the absorbance was quantified by measuring OD 568 using a microplate reader.

### Measurement of glucose uptake in cultured cells

Glucose Uptake Assay Kit (Ab83426, Abcam, Cambridge, UK) was used to measure the uptake of glucose by cultured NP cells. The assay was performed as per the manufacturer’s instructions. The concentration of glucose-6-phosphate in the experimental samples was calculated using the standard curve.

### ELISA assay

TNF-*α* and IL-1*β* are important inflammatory mediators associated with IVD degeneration. Human TNF-*α*,ELISA Kit(Elabscience, E-EL-H0109c) and Human IL-1*β* ELISA Kit (Elabscience, E-EL-H0149c) were used. Secreted pro-inflammatory cytokines in NP cells of different groups were detected by ELISA methods according to manufacture’s procedures.

### RT-PCR analysis

Quantitative RT-PCR was carried out using an ABI7900/ illumina eco sequence detection system. PCR products were visualized on an ethidium bromide stained agarose gel. The primers used were as Table 1.The cycle threshold (Ct) data were collected and normalized to *β*-actin. PCR products were electrophoresed in 6% polyacrylamide gels and visualized by ethidium bromide staining. Bio-Rad image system was used to quantify the results. Analysis of relative gene expression was calculated using the 2 (−Delta Delta C(T)) Method (2^−ΔΔC^).

### Protein extraction and western blot

Protein extracts were separated by 10–12% SDS-PAGE and transferred to PVDF membranes. After blocked with 5% non-fat milk, the membranes were incubated with primary antibodies (as shown in Table 2) at 4 °C overnight. The membranes were then incubated with the appropriate horseradish peroxidase-conjugated secondary antibody, anti-rabbit (1 : 5000; BA1054, Boster), anti-mouse (1 : 5000; BA1051,Boster), anti-goat (1 : 5000; BA1060, Boster), at room temperature for 1 h. The membranes were then washed in TBST buffer and protein bands were visualized with diaminobenzidine (DAB). The intensity of the bands was quantified and analyzed using Bio-Rad image software. *β*-Actin was used as loading standard.

### TUNEL apoptosis assay

The cells were fixed in 4% paraformaldehyde (pH 7.4) at room temperature for 25 min, washed thrice in PBS, and analyzed using *in situ* cell death detection kit (Roche, Basel, Switzerland) according to the manufacturer’s instructions. The slides were observed under fluorescence microscope.

### Immunofluorescence

Cells attached to slides were fixed with 4% paraformaldehyde and permeabilized with 0.2% Triton X-100 in PBS. After washing, the slides were blocked with 2% bovine serum albumin (BSA) in PBS for 2 h at 37 °C and then incubated with primary antibodies against: anti-IAPP (1 : 100),anti-caspase-3 (1 : 150). The cells were then incubated with appropriate secondary antibodies; goat anti-rabbit fluorescein-conjugated antibody (1 : 200) or goat anti-mouse fluorescein-conjugated antibody (1 : 200, Boster) for 1 h at room temperature. Nuclei were visualized by staining with DAPI (Beyotime Biotechnology, 0.5 g/ml). Slides were imaged using a microscope with appropriate filters (Olympus), and the images were analyzed using imaging software.

### Flow cytometric analysis

Apoptosis in cell cultures was quantitated by flow cytometry using Annexin V-APC/7-AAD Apoptosis Detection Kit (KeyGEN BioTECH (Jiangsu, China), KGA108). The fluorescence intensity of apoptotic cells was analyzed with a flow cytometer.

### Transmission electronic microscopy (EM) for autophagosomes

After deprivation, the cells were washed in 0.2% PBS and fixed % osmium tetroxide in water for 1 h. After that,the cells were stained in 2% uranyl acetate in darkness for 1 h. Then, the cells were dehydrated in ethanol against a concentration gradient and embedded in Durcopan ACM resin. Finally, the specimen were cut into thin sections and examined under a transmission electron microscope.

### Statistical analysis

All experiments were performed independently at least in triplicate, and the data are presented as mean±S.E.M. Statistical significance was determined by unpaired Student’s *t*-test or one-way ANOVA using GraphPad IPrism 5 software (La Jolla, CA, USA). *P*<0.05 was Statistical significance was set to *P*<0.05.

## Figures and Tables

**Table 1:** Primer sequence

***Name***	***Primer***	***Sequence***	***Size***
Homo b-actin	Forward	5′- AGCGAGCATCCCCCAAAGTT-3′	285 bp
	Reverse	5′- GGGCACGAAGGCTCATCATT-3′	
Homo IAPP	Forward	5′- GCTACACCCATTGAAAGTC-3′	102 bp
	Reverse	5′- GTTGTTGCTGGAATGAACT-3′	
Homo COL2A1	Forward	5′-A GAACTGGTGGAGCAGCAAGA-3′	142 bp
	Reverse	5′- AGCAGGCGTAGGAAGGTCAT-3′	
Homo COL1A2	Forward	5′- GCGGTGGTGGTTATGACTTTGGT-3′	176 bp
	Reverse	5′- TGTGCGAGCTGGGTTCTTTCTA-3′	
Homo Aggrecan	Forward	5′- TGAGCGGCAGCACTTTGAC-3′	287 bp
	Reverse	5′- TGAGTACAGGAGGCTTGAGG -3′	
Homo BG	Forward	5′- AAGGGTCTCCAGCACCTCTAC-3′	238 bp
	Reverse	5′- TCTCGATGCAGTTCATGTTCCGG-3′	
Homo casp3	Forward	5′- TGGTTCATCCAGTCGCTTTG-3′	100 bp
	Reverse	5′- ATTCTGTTGCCACCTTTCG -3′	
Homo calcr	Forward	5′- TGATTCATTTCCAGGGCTTCT-3′	240 bp
	Reverse	5′- TCTCCTCGCCTTGGTTGTT-3′	
Homo bcl2	Forward	5′- TGGTGGAGGAGCTCTT-3′	152 bp
	Reverse	5′- CCGGTTCAGGTACTCAGTCATC-3′	
Homo bax	Forward	5′- TCTGACGGCAACTTCAACTG-3′	188 bp
	Reverse	5′- TTGAGGAGTCTCACCCAACC-3′	
Homo beclin1	Forward	5′- CAATGGTGGCTTTCCTGGAC-3′	186 bp
	Reverse	5′- TGAGAGCTTTTGTCCACTGCT-3′	
Homo atg7	Forward	5′- GGTCAAAGGACGAAGATAACA-3′	148 bp
	Reverse	5′- GGTCACGGAAGCAAACAACT-3′	
Homo atg5	Forward	5′-CACAAGCAACTCTGGATGGGATT-3′	248 bp
	Reverse	5′- CCATCTTCAGGATCAATAGCAG-3′	
Homo RAMP1	Forward	5′- AATGCAGAGGTGGACAGGTT-3′	119 bp
	Reverse	5′- ACCACGATGAAGGGGTAGAGG-3′	
Homo RAMP2	Forward	5′-AGAGTTGTTTGACCTGGGCTTCC-3′	212 bp
	Reverse	5′-CCTGGGCCTCACTGTCTTTAC-3′	
Homo RAMP3	Forward	5′-TTCGCAGACATGATGGGCAAGGT-3′	258 bp
	Reverse	5′-CAGAACGACGGGTATAACGATCAG-3′	
Homo IL1b	Forward	5′- ATGGCTTATTACAGTGGCA-3′	139 bp
	Reverse	5′- TGTAGTGGTGGTCGGAGA-3′	
Homo TNF-a	Forward	5′- GCCTGTAGCCCATGTTGTAGCA-3′	163 bp
	Reverse	5′- CTTGAAGAGGACCTGGGAGTAG-3′	

**Table 2:** Antibodies for western blot analysis

**Primary antibody**	**Size**	**Company**	**Catalog No**	**Dilution**
Anti-rabbit GAPDH	37 kD	GOOD HERE, Hanzhou	AB-P-R 001	1 : 1000
Anti-rabbit RAMP3	16 kD	Abcam	ab78017	1 : 500
Anti-rabbit CALCR	56.9 kD	Abcam	ab11042	1 : 500
Anti-rabbit LC3	14, 16 kD	CST	2775	1 : 1000
Anti-rabbit AKT	60 kD	ProteinTech Group	10176-2-AP	1 : 1000
Anti-rabbit P-AKT	60 kD	CST	4060P	1 : 2000
Anti-rabbit mTOR	289 kD	Bioworld	BS3611	1 : 800
Anti-rabbit P-mTOR	289 kD	Bioworld	BS4706	1 : 800
Anti-rabbit mmp3	60 kD	CST	14351	1 : 1000
Anti-rabbit mmp9	92 kD	Bioworld	BS6893	1 : 1000
Anti-rabbit mmp13	54 kD	Abcam	ab39012	1 : 4000
Anti-rabbit ADAMTS4	90 kD	Elabscience	EAP1002	1 : 1000
Anti-rabbit ADAMTS5	102 kD	Abcam	ab135656	1 : 80
Anti-mouse Aggrecan	50–60 kD	Abcam	ab3778	1 : 100
Anti-goat colII	190 kD	Santa	SC-7764	1 : 500
Anti-rabbit P38	41 kD	ProteinTech Group	14064-1-AP	1 : 800
Anti-rabbit P-P38	43 kD	CST	4511P	1 : 1000
Anti-rabbit JNK	54, 46 kD	CST	9252	1 : 1000
Anti-rabbit P-JNK	46, 54 kD	CST	4668P	1 : 1000
Anti-rabbit PI3K	85 kD	CST	4257P	1 : 1000
Anti-goat P-PI3K	85 kD	Santa	SC-12929	1 : 500
