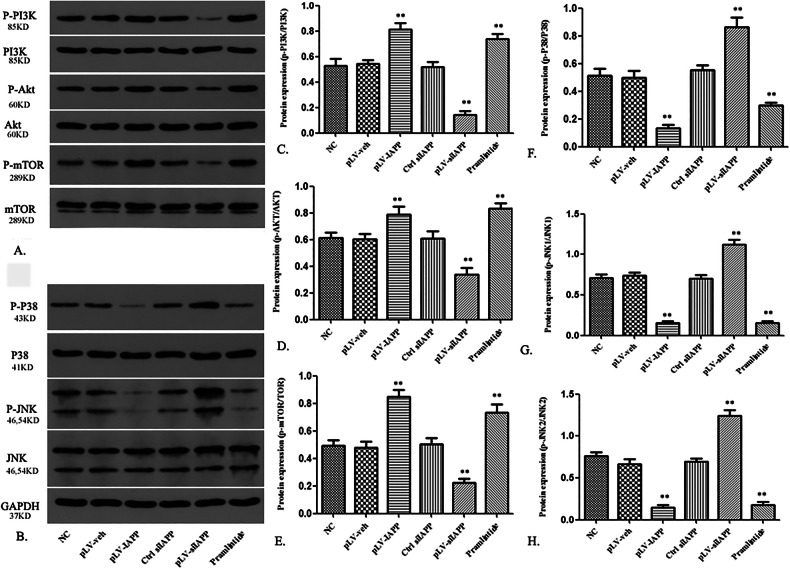# Correction to: IAPP modulates cellular autophagy, apoptosis, and extracellular matrix metabolism in human intervertebral disc cells

**DOI:** 10.1038/s41420-025-02562-1

**Published:** 2025-07-25

**Authors:** Xinghuo Wu, Yu Song, Wei Liu, Kun Wang, Yong Gao, Shuai Li, Zhenfeng Duan, Zengwu Shao, Shuhua Yang, Cao Yang

**Affiliations:** 1 Department of Orthopaedic Surgery, Union Hospital, Tongji Medical College, Huazhong University of Science and Technology, Wuhan, 430022 China; 2 Department of Orthopaedic Surgery, Massachusetts General Hospital and Harvard Medical School, Boston, MA USA

Correction to: *Cell Death Discovery* 10.1038/cddiscovery.2016.107, published online 30 January 2017

During our inspection of the manuscript, we noticed an inadvertent reuse error in Fig. 1a (in the panel of Alcian blue staining). This occurred due to an oversight during figure assembly. We conducted a series of Alcian blue stainings on degenerated intervertebral disc tissues to represent pathological changes associated with different grades of degeneration. These stained images were provided as supporting evidence for another paper from our research team. This error does not affect the overall conclusions of the study but requires correction to ensure accuracy in data representation.

Originally published Fig. 1a
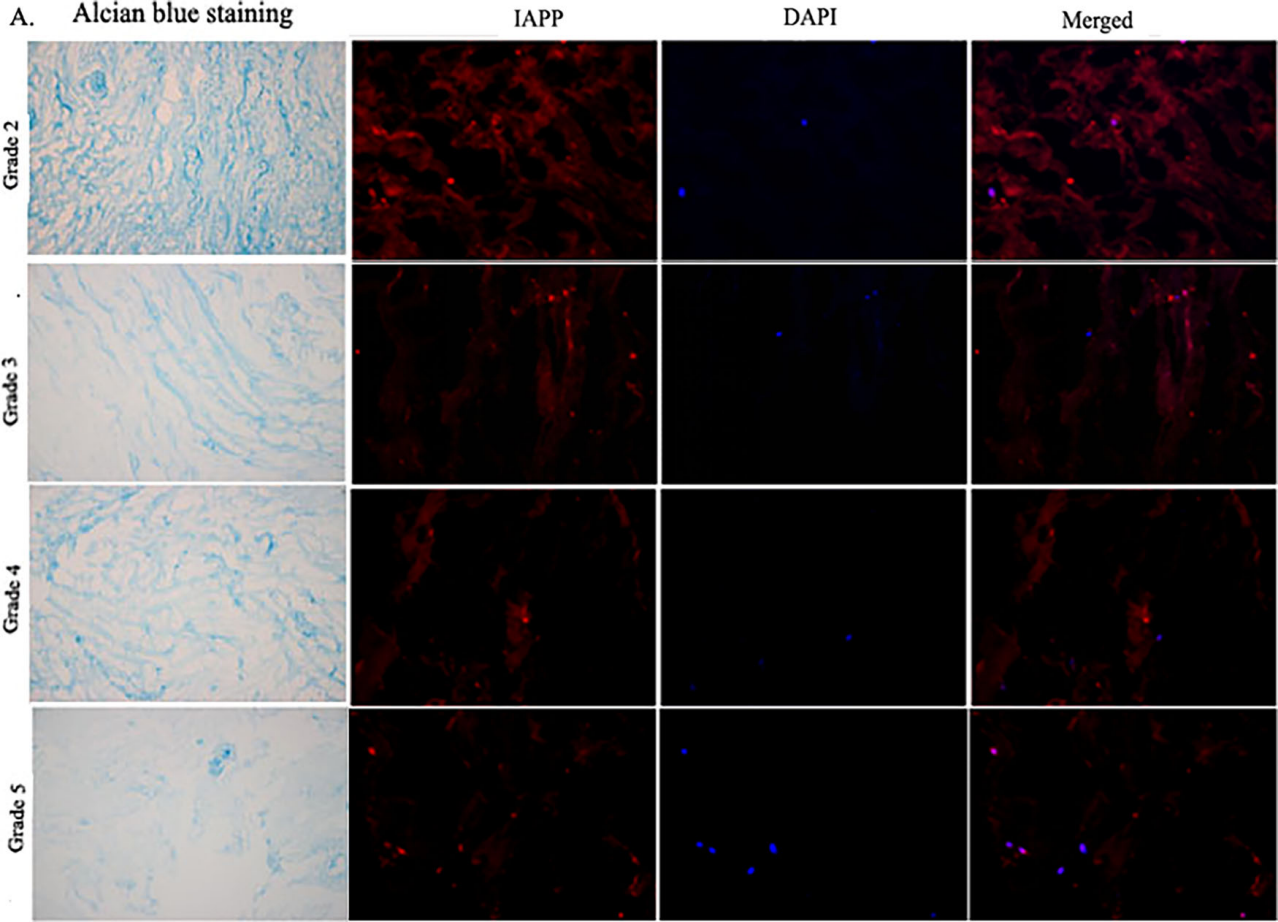


Revised Fig. 1a
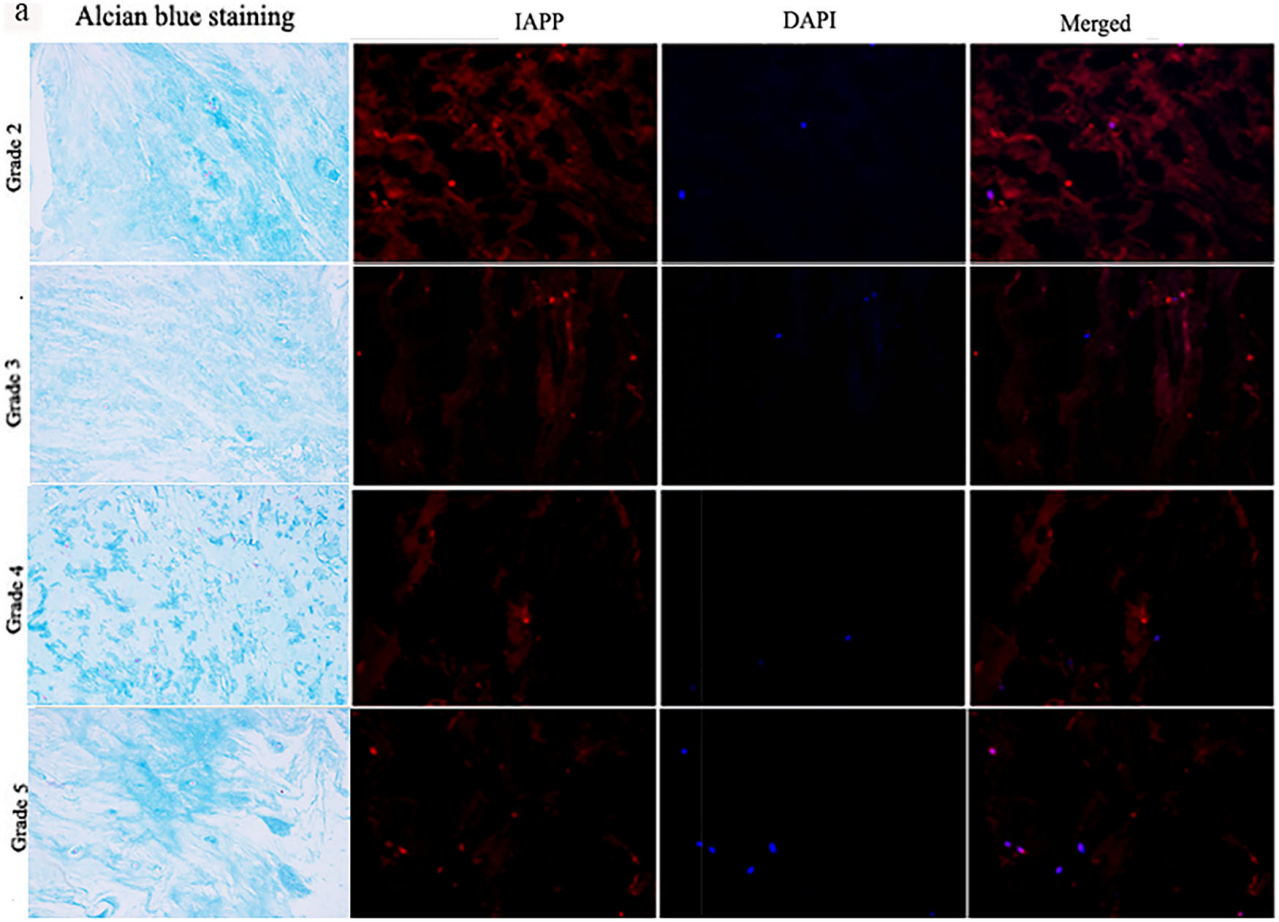


Figures 8A and 8B are Western blot results detecting the target protein and the loading standard (GAPDH) on the same membrane. When preparing the images originally, we included the loading standard in both panels A and B for visual symmetry, although this was not necessary. To avoid unnecessary confusion for readers and to meet strict inspection standards, we suggest removing the loading standard (GAPDH) from panel A and retaining it only in panel B.

Originally published Fig. 8
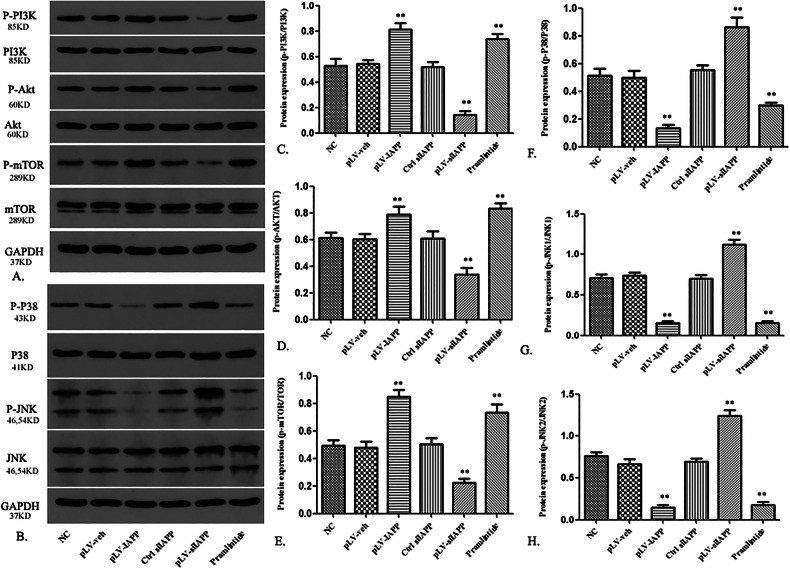


Revised Fig. 8